# Performance evaluation and relevance of the CellaVision™ DM96 system in routine analysis and in patients with malignant hematological diseases

**DOI:** 10.1111/j.1751-553X.2007.00996.x

**Published:** 2008-12

**Authors:** E CORNET, J-P PEROL, X TROUSSARD

**Affiliations:** * Laboratoire d’hématologie, CHU Côte de NacreCaen, France; † Sysmex Europe GMBHNorderstedt, Germany

**Keywords:** Hematology analyzer, DM96, automation

## Abstract

The CellaVision™ DM96 is an automated image analysis system dedicated to locating and preclassifying the various types of white blood cells in peripheral blood smears. The system also partially characterizes of the red blood cell morphology and is able to perform platelet counts. We routinely analyzed the blood samples from 440 patients with quantitative and/or qualitative abnormalities detected by the XE-2100 Sysmex™. Only 2.6% of cells are not identified by DM96™. After classification of the unidentified cells very good correlation coefficients are observed between DM96™ and manual microscopy for most hematological parameters and accuracy is judged excellent up to 98%. For most common parameters, false positive and false negative ratios are also very good. Whatever the pathology and the number of blasts on smear, all patients were positive for blast detection on DM96™. The system is a useful tool for assisting in the diagnosis and classification of most acute or chronic leukemia. Automatic cell location and preclassification, along with unique cell views on the computer screen, could reduce the time spent performing differentials and make real-time collaboration between colleagues a natural part of the classification process. The workstation also provides an ergonomically correct and relaxed working environment. We suggest its use in routine analysis; the system could be very helpful for the accurate morphological diagnosis of samples from patients with malignant hematological disease.

## Introduction

In recent years, there have been rapid developments in new techniques for the optimal management of patients with malignant hematological disease. Despite the increased contribution of immunological, cytogenetical and molecular investigations, morphological examination remains the first step for rapid and accurate diagnosis and optimal follow-up of patients with malignant blood diseases. This step requires a high quality stained blood smear preparation for the accurate assessment of cellular morphology. Despite the significant improvements during the last years in hematology analyzers, no significant progress has been made in terms of automatic examination of peripheral blood cells. Irrespective of the analyzer, approximately 15% of the blood samples require manual microscopic observation either because of biological rules or analyzer flags. The relative number of samples to be reviewed will probably not decrease in years to come. Smear examinations are time consuming and require well-trained medical technologists and biologists.

Microscopy automation should be available in hematology laboratories. The decrease of cytology proficiency in the daily practice, the need for development of new innovative techniques in hematology laboratories in the face of limited human resources, and finally, the increase and the complexity of pathologies attributable to population aging create a need for automation of the cytology platform in all laboratories. In this context, we had the opportunity to evaluate the CellaVision™ DM96 automated microscope (CellaVision AB, Ideon Research Park, SE-223, 70 Lund, Sweden) in the hematology laboratory at Caen University Hospital. The academic hospital has 1750 active beds and the laboratory performs 500–600 Complete Blood Count with Differential (CBC-DIFF) per day with XE-2100 Sysmex™ analyzers (Sysmex Corporation 1-S-1, Wakinohama Kaygandori, Cho-Ku, Kobe 651-0073, Japan). Adult and pediatric hematology account for 10% of the demands, oncology represents 15% and surgical and intensive cares about 20%. We evaluated CellaVision™ DM96 and discuss how such a device could be integrated into the daily routine and the performance of DM96™ in the diagnosis and monitoring of patients with malignant hematological diseases.

## Materials and methods

### The automated microscope DM 96

**Figure 1:**
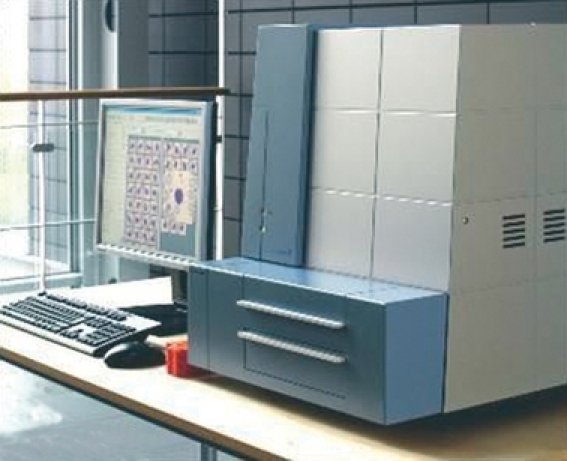
DM96™: an automated device for the differential counting of white blood cells (WBCs).

**Figure 2:**
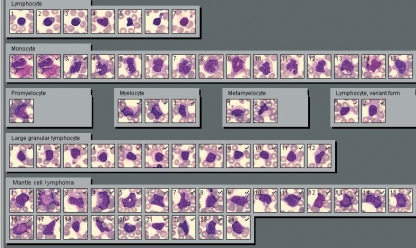
DM96™ performs the acquisition and preclassification of cells and the operator subsequently verifies and modifies, if necessary, the suggested classification of each cell.

CellaVision™ DM96 is an automated device for the differential counting of white blood cells (WBCs) and characterization of red blood cells (RBCs). It consists of a slide feeder unit, a microscope with three objectives (×10, ×50, and ×100), a camera and a computer system containing the acquisition and classification software CellaVision™ blood differential software (Figure 1). A slide autoloader facilitates the automatic analysis of up to 96 smears with continuous loading access. The number of WBC to be analyzed is user definable from 100 up to 400. To perform a differential count, a thin film of blood is wedged on a glass slide (a blood smear) from a peripheral blood sample and stained according to the May-Grunwald Giemsa protocol. The analyzer performs the acquisition and preclassification of cells and the operator subsequently verifies and modifies, if necessary, the suggested classification of each cell (Figure 2). The operator can also introduce additional observations and comments when needed. For this reason, persons specially trained in the use of this instrument and skilled in the recognition of cells can operate the DM96™. The system makes the following WBC classifications: band neutrophils, segmented neutrophils, eosinophils, basophils, monocytes, lymphocytes, promyelocytes, myelocytes, metamyelocytes, blast cells, variant form lymphocytes and plasma cells. The system also preclassifies non-WBC into the following classifications: erythroblasts, giant thrombocytes, thrombocytes aggregation, smudge cells and artifacts. ‘Unidentified’ is a class of cells and objects that the system cannot identify. The system has four flag levels for the following RBC morphological characteristics: polychromasia, hypochromasia, anisocytosis, microcytosis, macrocytosis, and poikilocytosis. Besides the WBCs mentioned above, the operator or ‘user’ can reclassify cells into the following classes afterwards: immature eosinophils, immature basophils, promonocytes, prolymphocytes, large granular lymphocytes, hairy cells, Sezary cells, others, megacaryocyte, not classed, and 15 user-defined classes. The operator can also add the following characteristics for RBCs: schizocytosis, helmet cells, sickle cells, spherocytosis, elliptocytosis, ovalocytosis, teardrop cells, stomatocytosis, acantocytosis, and echinocytosis, Howell–Jolly bodies, Pappenheimer bodies, basophilic stippling, parasites, and 10 other definable characteristics.

### Smears and stains

Slides analyzed by the DM96™ were prepared with SP-100 SYSMEX™ from venous blood sample collected in EDTA-type anticoagulant and previously analyzed with XE-2100 SYSMEX™. Staining program and reagents were as follows: May Grunwald (MG) and Giemsa (Biolyon, France), MG pure time: 2.5 min, MG dilute time: 3 min, Giemsa time: 7 min, rinse 0 min and drying time 5 min.

### Patients

Four hundred and forty nonselected patients processed with the XE-2100 were analyzed by medical technologists experienced using both conventional microscopy method and DM96™. All these samples were abnormal according to routine laboratory criteria and hence, justified a manual smear review (quantitative abnormality, qualitative flag from XE-2100™, malignant hematological disease). Under the microscope, 100 leucocytes were observed for establishing the control differential and a mean of 110 leucocytes were required for DM96™.

To analyze the performance of automated microscopy with DM96™ and measure its impact on laboratory organization and workflow, we studied its ability to correctly identify blood cells and accuracy compared with manual method and/or XE-2100™. Finally, we analyzed the sensitivity for detection of pathological cells in case of hematological disease.

### Efficiency of cell recognition

We analyzed the accuracy in classifying normal and abnormal cells for routine parameters by DM96™ (neutrophils, eosinophils, basophils, lymphocytes, monocytes, immature granulocytes, and erythroblasts) out of 62904 cells [including Nucleated Red Blood Cells (NRBCs) and smudge cells] issued from the 440 patients analyzed. Efficiency of recognition has been calculated for each cell category. Unidentified cells from DM96™ have been studied as well and their influence on the above-mentioned result calculated.

### Comparison with manual method

We did not test normal blood samples in this study as this had already been performed and the results showed DM96™ to be reliable and accurate (Ceelie, Dinkelaar & van Gelder, 2007). The results of 356 patients with no hematological disease but laboratory flagging criteria obtained on DM96™ were compared after medical technologist reclassification with the manual differentials performed by the same user and to XE-2100™. Correlation between DM96™ and both manual count and XE-2100™ result was established for neutrophils, eosinophils, basophils, lymphocytes, monocytes, erythroblasts, and immature granulocytes (including metamyelocytes, myelocytes, and promyelocytes). In case of disagreement, a clinician reanalyzed both the slide and the validation issued from DM96™.

### Malignant hematological diseases

We focused then on 84 patients with malignant hematological disorders from various types. The classification of these 84 patients was made according to the WHO criteria (Harris *et al.*,1999) and is described in table 1. Blast recognition and quantification by DM96™ was studied in 34 patients, acute lymphoblastic leukemia (ALL), acute myeloid leukemia (AML) or chronic myeloproliferative disorders/myelodysplastic syndromes (CMPD/MDS). Three patients were excluded for the analysis in the absence of blasts cells in the peripheral blood. All these three patients had myelodysplasic syndromes (MDS). For all other patients, B-cell chronic lymphocytic leukemia (CLL), other B-cell chronic lymphoproliferative disorders (B-CLPD), we focused on capacity of DM96™ to efficiently recognize mature cells and provide images permitting an easy and reliable morphological classification.

**Table 1:** Distribution of the hematological diseases in 84 patients

Acute myeloid leukemia (AML)	17
Acute lymphoid leukemia (ALL)	10
Myeloproliferative/myelodysplastic syndromes(MPD/MDS)	10
B-cell chronic lymphocytic leukemia (CLL)	25
Other B-cell chronic lymphoproliferative disorders	22
Mantle cell lymphoma (MCL)	5
Follicular lymphoma (FL)	1
Diffuse large B-cell lymphoma (DLBCL)	7
Hodgkin lymphoma (HL)	1
Splenic lymphoma with villous lymphocytes (SLVL)	3
Hairy cell leukemia (HCL)	1
Persistent polyclonal B lymphocytosis (PPBL)	3
Large granular lymphocytes leukemia (LGL)	1

### Statistical analysis

Statistical analysis was performed using Microsoft® Excel software. For correlation analysis, we used two-tailed paired *t*-tests to evaluate differences between the percentage of blast cells detected by DM96™ and manual microscope in patients with blast cells in the peripheral blood. Clinical sensitivity and specificity of the CellaVision DM96™ were defined as its ability to obtain positive and negative results concordant with medical technologist before and after classification of unidentified cells by DM96™.

## Results

### Accuracy of cell recognition

Only 2.6% of cells are not identified (especially NRBCs and immature granulocytes) leading the global efficiency of DM96™ to 95% of direct correct identification. In total, when reclassifying unidentified cells by medical technologist, accuracy is judged excellent up to 98%. For most common parameters, false positive and false negative ratio are very good (Table 2).

**Table 2:** Comparaison between DM96™ before and after classification of unidentified cells and manual differential counts

Cell per cell analysis with DM 96: pool of 62 904 cells								
DM96™\user	Neutrophils	Eosinophils	Basophils	Lymphocytes	Monocytes	IG	NRBC	total
Before unidentified cells classification								
Unidentified	796	36	9	56	214	353	157	1621
Accuracy (%)	95.6	96	80	99	92	58	56	95
After unidentified cells classification (%)								
Accuracy	98	98	83	99	98	86	82	98
False negative	2.3	1.5	17.3	0.5	2.4	14.4	17.9	
False positive	0.0	12.3	39.3	2.2	4.9	46.2	6.3	

IG, Immature Granulocytes; NRBC, nucleated red blood cell.

### Comparison with manual method

Correlation for DM96™ results with the manual method and/or XE-2100™ is excellent for neutrophils, lymphocytes, eosinophils, and acceptable for immature granulocytes, erythroblasts, and for basophils. The correlation observed for monocytes was not as good as expected; both results (DM96™ and optical manual count) were usually lower than the automatic count but this was not clinically relevant.

### Patients with malignant hematological diseases

#### Patients with blasts on smear

**Figure 3:**
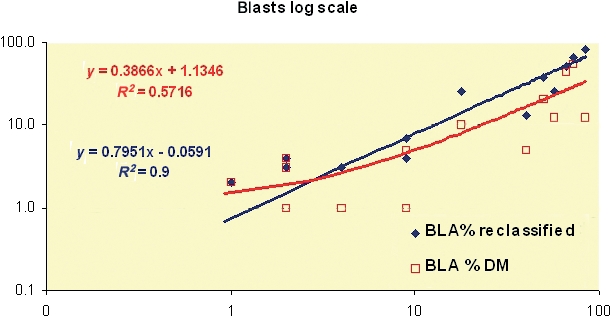
Blasts log scale.

Whatever the pathology (AML, ALL, and CMPD/MDS) and the number of blasts on smear, all 34 patients were positive for blast detection on DM96™. Additionally, it appears very easy to distinguish myeloid blasts from lymphoid blasts. Despite DM96™ underestimates the number of blasts cells (especially in ALL where a huge number of them is misclassified as lymphocytes), after manual validation, the correlation with microscope appears very good (Figure 3). In patients with chronic myeloproliferative disorders or myelodysplastic syndromes, both the quantitative and qualitative analysis of immature granulocytes was comparable with the one observed for routine patients. Basophiles were clearly identified even when they show abnormal aspects. Blast count for these patients appeared to be reliable.

#### B-cell chronic lymphoproliferative disorders

**Figure 4:**
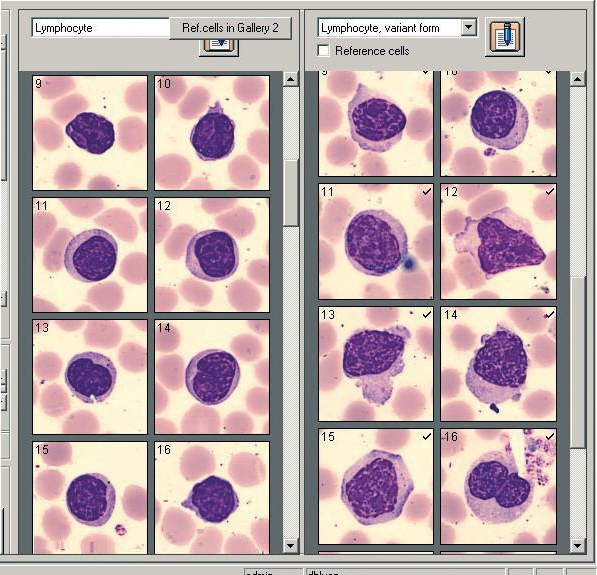
The overview of tumoral lymphoid cells facilitates the efficient distinction between typical chronic lymphocytic leukemia and other B-cell chronic lymphoproliferative disorders.

DM96™ classifies CLL cells without problem but often provides a wide count of ‘smudge cells’ arising from the smear method. The recommended procedure in this case is still to use the lymphocyte count from the analyzer as the most reliable result. Concerning other lymphoid pathologies summarized in Table 1, as the DM96™ only gets capacities for 4 cells groups (lymphocytes, variant lymphocytes, plasma cells, blasts) it is not able to classify correctly these abnormal samples. Such classification remains under the responsibility of medical technologist. Observation of lymphoid cells on DM96™ screen allowed an easy and accurate identification of abnormal cells such as large granular lymphocytes (LGL), binucleated lymphoid cells and hairy cells, which are not expected to be classified directly by the DM96™ supports to clearly identify the exact disease under consideration [splenic lymphoma with villous lymphocytes (SLVL), follicular lymphoma, mantle cell lymphoma; Figure 4].

## Discussion

The DM96™ has already proven to be reliable for normal patient samples. In this study, we became aware of the capabilities and limitations of the automated microscope DM96™ for analyzing patient samples with quantitative and qualitative abnormalities detected by XE-2100™. From a biological point of view, we as others (Swolin *et al.*, 2003; Kratz *et al.*, 2005; Roumier *et al.*, 2005; Contis & Williams, 2006, 2006) demonstrated that the results obtained from DM96™ correlated well to those obtained by manual counting of all patient samples, suggesting that DM96™ may be useful for the analysis of the great majority of parameters tested. About monocytes, heterogeneous distribution on slides is a well-known problem arising directly from the smear method and consequently, both the choice of the observation area (which can be different for DM96™ and manual microscopy) and the number of cells analyzed contribute to the difference in monocyte counts.

Modern characterization of acute malignant hematological disease is a multidisciplinary process. Initially, it requires the integration of clinical, morphological and cytochemical information. A correct and rapid hematological evaluation is necessary to follow-up with the appropriate laboratory tests, specifically immunophenotyping, metaphase cytogenetics and molecular studies. However, the interpretation of morphological and cytochemical stains remains central to the diagnosis and classification of AML. The identification of multilineage dysplasia is entirely dependent on light microscopic assessment of the leukemia cells. Despite the advances in diagnostic technologies, the maintenance and improvement of morphological skills still remain essential requirements in the diagnosis of AML. In case of AML, DM96™ is able to detect blast cells and to identify myeloid blast cells and maturing cells. In addition, it facilitates the evaluation and quantification of dysplasia on a high number of myeloid cells preselected by DM96™, which are then classified and eventually properly reclassified by the biologist. In cases of B-ALL and more generally in cases of myeloperoxydase (MPO) negative blast cells, immunophenotyping is always still required for the initial diagnosis. DM96 is also able to detect blast cells but is unable to classify blast cells as lymphoblasts. In this context, it makes sense to rely upon conventional microscopy. If the sample quality is poor, it will be also necessary to survey the entire smear in manual mode. For post-treatment monitoring of patients with malignant hematological disease, DM96™ represents a good tool for the detection of abnormal cells but is not appropriate for quantifying blast cells in the peripheral blood of patients with B-ALL. In cases of B-CLPD and when operated by an experienced cytologist, DM96™ is helpful for identifying the disease and especially the lymphoid abnormalities. As an example, we rapidly identified binucleated lymphocytes characteristic of polyclonal lymphocytosis with binucleated lymphocytes (Mossafa *et al.* 1999), hairy cells in patients with HCL and atypical lymphocytes in patients with B-CLL. An overview of all lymphoid cells is of great interest in lymphocyte analysis.

Finally, DM96 proved to be fully comparable with the manual method in a test using control patient blood smears and in daily practice applicable to >90% of the leucocytes review.

The validation and screening of abnormal smears is one of the core competencies of the technical staff, which is under the supervision of a biologist. For such a routine process, a significant timesaving could be realized by implementing such a reliable automatic system. Therefore, these observations should provide food for thought when considering modalities for improving the efficiency of a hematology laboratory.

The routine introduction of DM96™ will probably have a great impact on the logistics and organization of both specialized and general hematology laboratories. Depending on the validation requirements and guidelines in each country, all the smears performed by SP-100™ can be passed onto the DM96™ in a continuous mode. If, after the unidentified cells have been identified, classified, confirmed and validated, there are no blasts present, the validation could be carried out by the DM96™, except when particular difficulties are encountered. For the technical staff, the installation of DM96™ would have many consequences: the reduction of technical staff time at the microscope while simultaneously increasing the efficiency of the workflow, the elimination of medical technologists facing a difficult diagnosis alone, improved ergonomics of the workstation (elbows, eyes, and back), reduction of the filing of the blades and finally, optimization of time and quality. The introduction of DM96™ could also optimize the time of the biological staff and improve the proficiency of morphological expertise. In addition, the easy and clear presentation of all patient samples on a computer screen will help ensure the quality of follow-up care in cancer patients. The images can be transmitted to other experts for consultation and confirmation and will facilitate validation of clinical protocols. We also can hope for shorter response times, a reduction in errors, an improvement in continued and advanced education, possibly a redeployment of human resources and a significant cost reduction for a hemogram.
